# Supplementary material for: Antagonistic behavior of brain networks mediated by low-frequency oscillations: electrophysiological dynamics during internal–external attention switching
Source: Commun Biol. 2024 Sep 9;7:1105. doi: 10.1038/s42003-024-06732-2 (PMC11385230; doi:10.1038/s42003-024-06732-2)
Supplement: Supplementary file 1 — Supplementary information [file 42003_2024_6732_MOESM1_ESM.pdf]

Supplementary Information

Patient No.	Age (years)	Gender	Handedness	Suspected seizure onset zone	Epilepsy duration (years)
P1	31	M	R	Left frontal lobe–premotor area	2
P2	26	F	R	Right frontal lobe, insula	13
P3	38	F	R	Right insula, operculum	9
P4	41	F	R	Right frontal lobe–prefrontal area	32
P5	15	M	R	Left frontal and temporal lobe	7
P6	16	M	L	Left insula	11
P7	44	F	R	Right parietal lobe	31
P8	48	F	R	Right temporal lobe	31
P9	12	F	R	Left frontal lobe–prefrontal area	9
P10	15	F	R	Left temporal lobe	2
P11	43	M	R	Right temporal, parietal, and occipital lobes	34
P12	46	M	L	Left frontal lobe	34
P13	29	F	R	Right frontal and temporal lobe	20
P14	35	F	L	Left temporal lobe	21
P15	55	M	R	Right frontal lobe	27
P16	46	F	R	Right frontal lobe–medial cortex	44
P17	37	F	L	Right temporal lobe	13
P18	44	M	R	Left frontal lobe–premotor area	36
P19	36	M	R	Right temporal lobe	12
P20	38	F	R	Right frontal lobe	20
P21	32	M	L	Right frontal lobe–orbitofrontal area	23
P22	28	F	R	Right frontal lobe	10
P23	24	F	L	Right temporal lobe	6
P24	49	M	R	Right temporal lobe	27
P25	30	F	L	Left temporal lobe	19

Supplementary Table 1. Subject information. F–female, M–male, R–right, L–left.

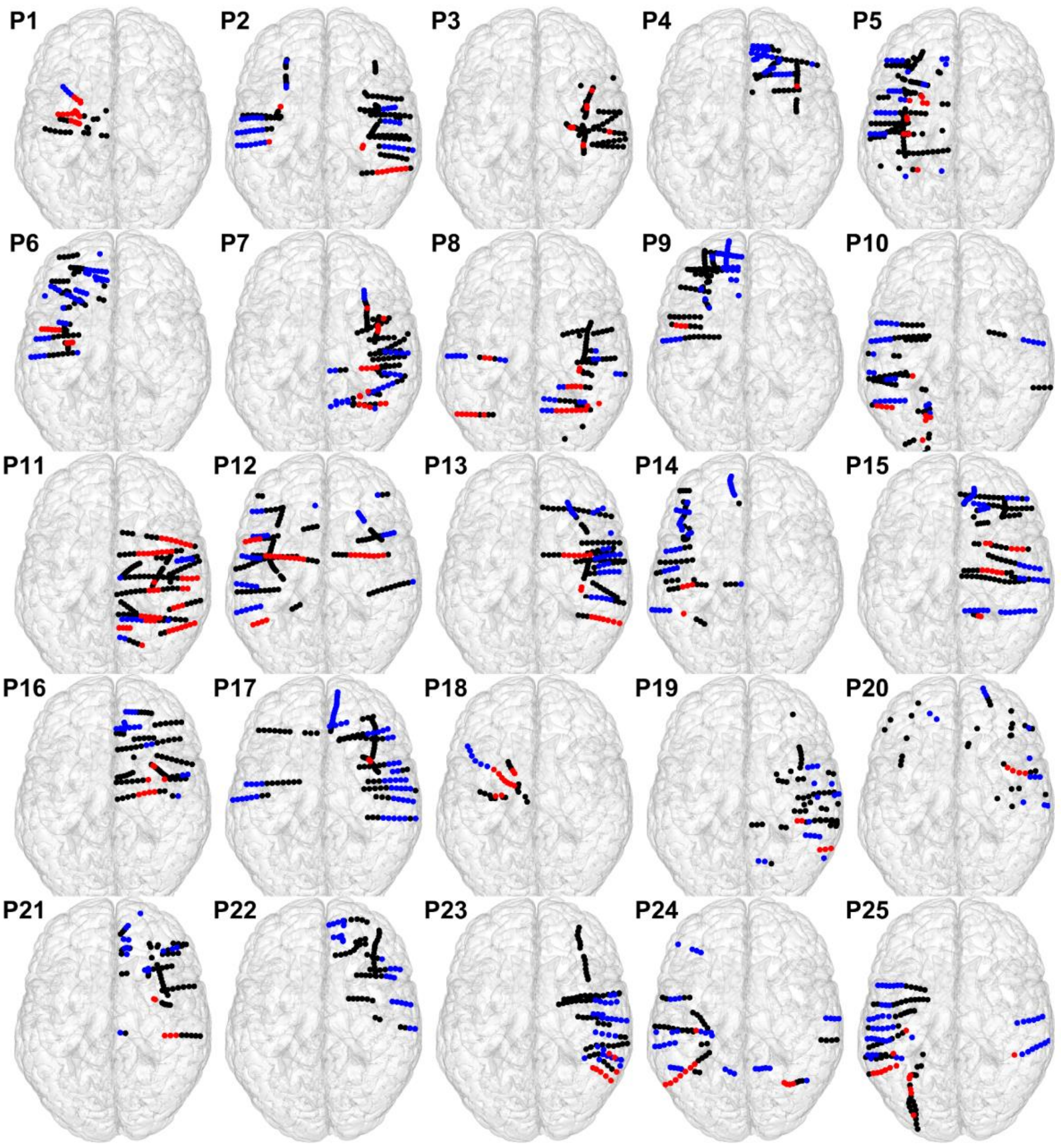

Supplementary Figure S1. Single-subject (P1–P25) iEEG implantation schemes. Top views of the MNI brain template (colin27). The colored circles represent the iEEG channels (i.e., the centers of the bipolar-referenced SEEG contact pairs). Note that (1) the iEEG channels were projected up front for visualization purposes (in reality they were located deep inside the brain); (2) some electrodes may appear curved due to MNI normalization (in reality, they were straight); and (3) the size of the circles does not match the physical size of the contacts, which were much smaller (cylindrical shape: 0.8-mm diameter and 2-mm height). The iEEG channels (black circles) were assigned to the DMN (blue) or the DAN (red). Although the implantations were very heterogeneous among subjects, 24 of them had at least some channels in both networks.

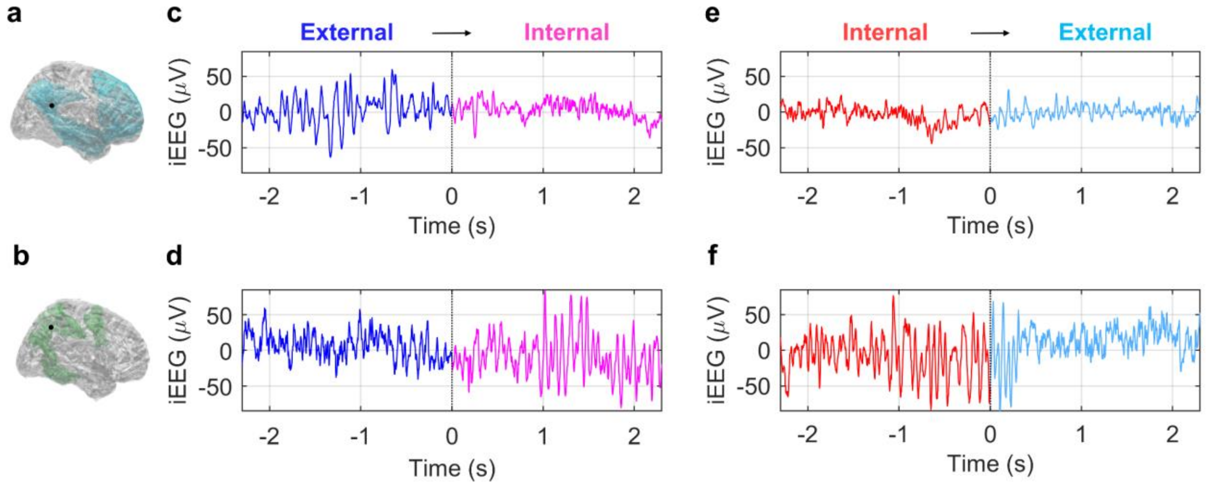

Supplementary Figure S2. Exemplary single trials of raw iEEG in the DMN and DAN. Two trials (E-I and I-E) measured simultaneously by two selected channels (from the DMN and DAN, the same channels as in Fig. 2) from subject P8 were selected for an illustration. **(a)** Right lateral right view of the brain template with a highlighted channel position (black dot) and DMN (cyan). **(b)** Same as in A, but for a channel in the DAN (in green). **(c)** A raw iEEG trace of a single trial recorded from the DMN channel during external–internal attention switching. **(d)** The same trial but recorded from the channel located in the DAN. **(e, f)** Another single trial during internal-external attention switching. The same notations as in c and d. The DMN was deactivated during the E-task (the iEEG oscillatory activity was dominated by slower frequency rhythms) and activated during the I-task (the iEEG activity was dominated by faster frequency rhythms). At the same time, the opposite situation was observed in the DAN (activated during the E-task and deactivated during the I-task).

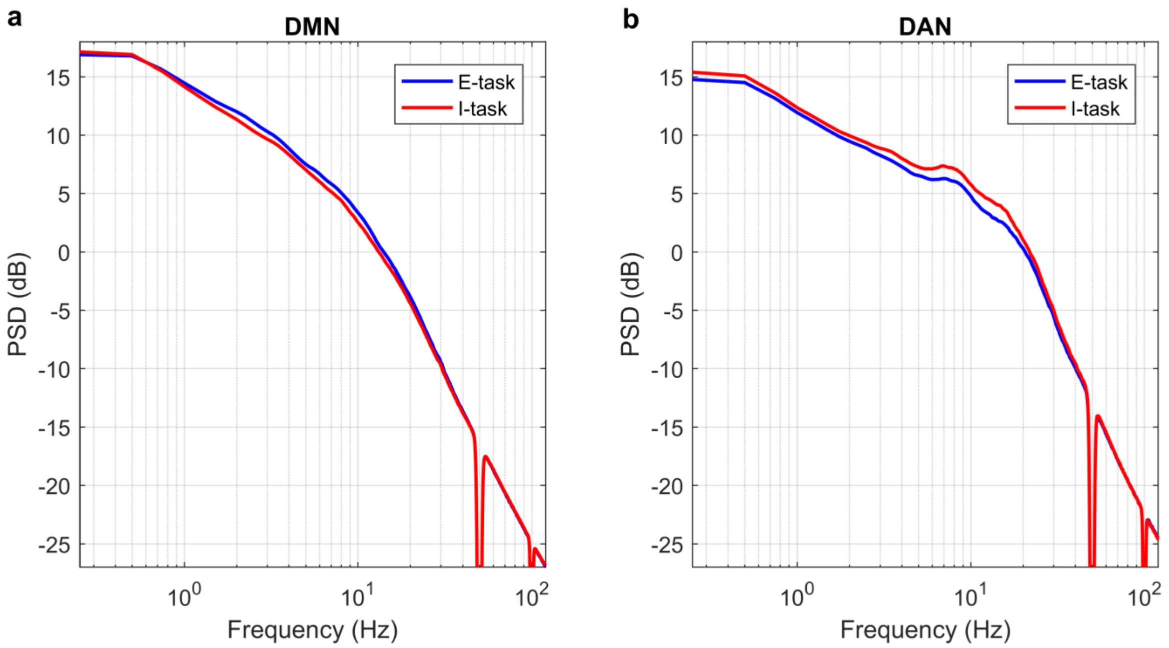

Supplementary Figure S3. The PSD of the attention-switching trials. The iEEG PSD was computed by Fourier transformation for the trials of the external (E-task, blue curves) and internal (I-task, red) attention switching tasks. The duration of the trials was 2.3 s (either before or after the task switch) Note the log-log scale of both frequency (x-axis) and the PSD (y-axis), resulting in the typical linear drop of PSD with increasing frequency. The dips in the PSD correspond to the effects of the 50-Hz (and 100-Hz) notch filters. All channels assigned to the **(a)** DMN (741 channels, 24 subjects) or **(b)** DAN (297 channels, 24 subjects) were used for the PSD computation. There was a slight increase of the external attention task in the low-frequencies as compared to the internal attention task in the DMN (and vice versa for the DAN).

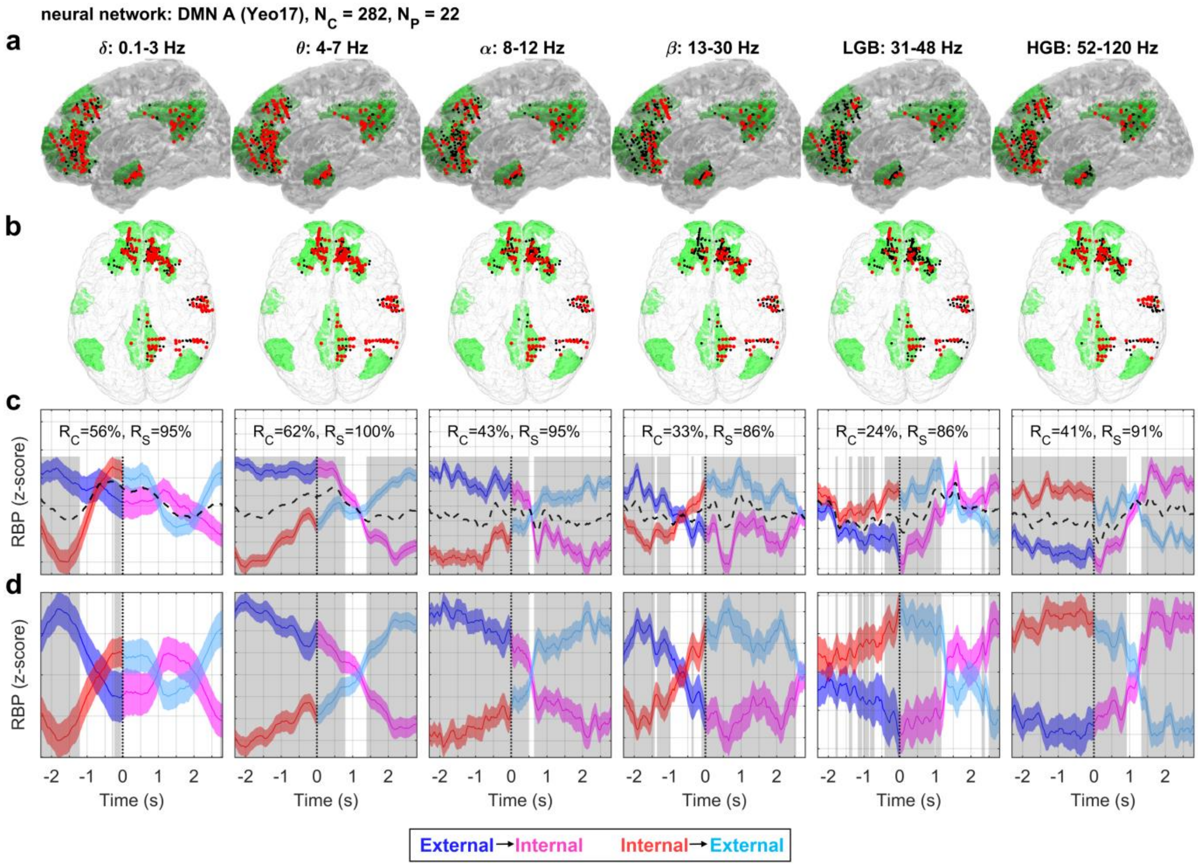

Supplementary Figure S4. The DMN-A during the attention-switching task. Same notations as in Fig. 4. The DMN-A (based on the Yeo-17 brain parcellation, highlighted in green) consisted of 282 channels in 22 subjects. The channels were anatomically distributed mostly in the frontal lobe (anterior cingulate cortex, middle frontopolar gyrus, superior and middle frontal gyrus, and rostral gyrus) but also in the temporal lobe (middle temporal gyrus) and parietal lobe (angular gyrus and precuneus). Consistent with overall DMN behavior, there was a pronounced difference in the LFBs (< 30 Hz), especially in the alpha band (8–12 Hz), and we observed the opposite activation in the HGB. The relative decrease of the LFB power and increase in the HGB during the I-task and subsequent switch of the activation during the E-task, suggest DMN-A activation for the I-task (red and magenta curves) and deactivations/suppressions during the E-task (blue and cyan curves).

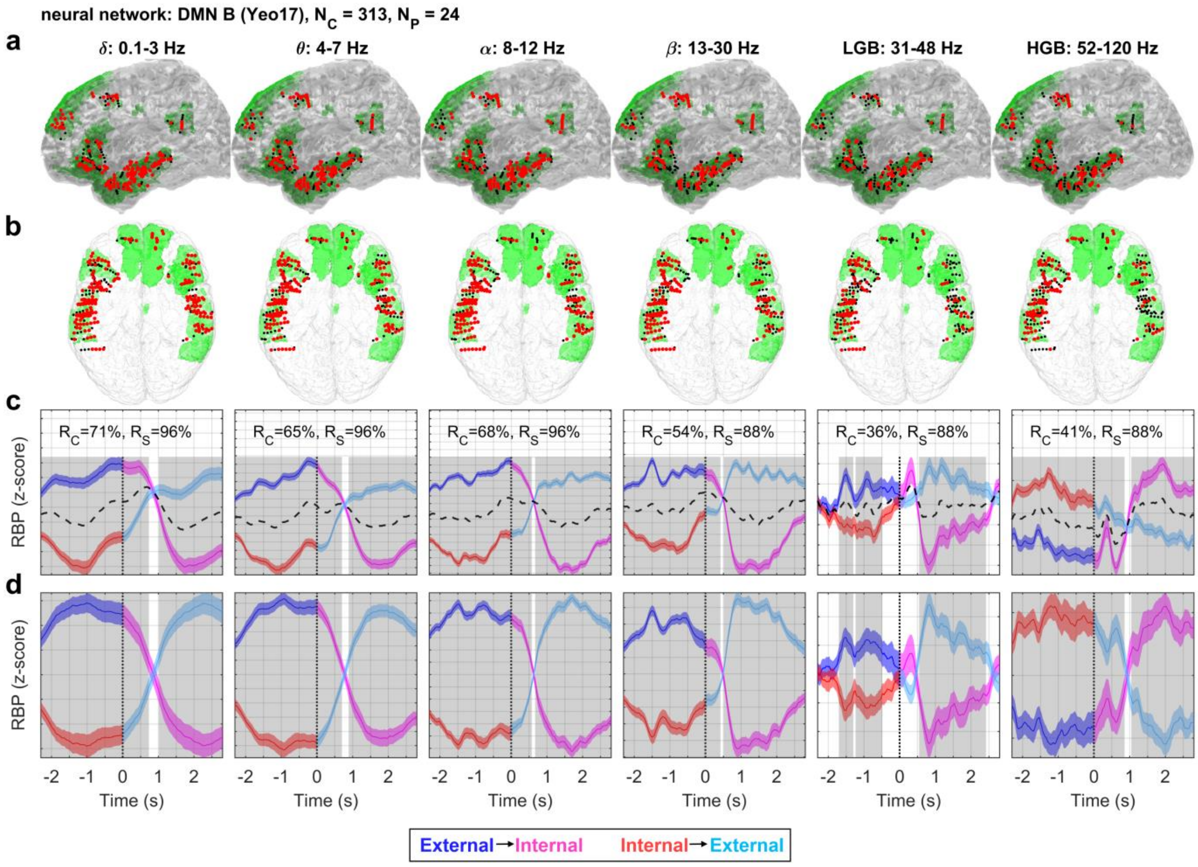

Supplementary Figure S5. The DMN-B during the attention-switching task. Same notations as in Fig. 4. The DMN-B (based on the Yeo-17 brain parcellation, highlighted in green) consisted of 313 channels in 24 subjects. The channels were anatomically distributed mostly in the temporal lobe (middle and superior temporal gyrus) but also in the frontal lobe (superior and inferior frontal gyrus) and parietal lobe (angular gyrus). Consistent with overall DMN behavior, there was a pronounced difference in the LFBs (< 30 Hz), especially in the alpha band (8–12 Hz), and we observed the opposite activation in the HGB. The relative decrease of the the LFB power and increase in the HGB during the I-task and subsequent switch of the activation during the E-task, suggest the DMN-B activation for the I-task (red and magenta curves) and deactivations/suppressions during the E-task (blue and cyan curves).

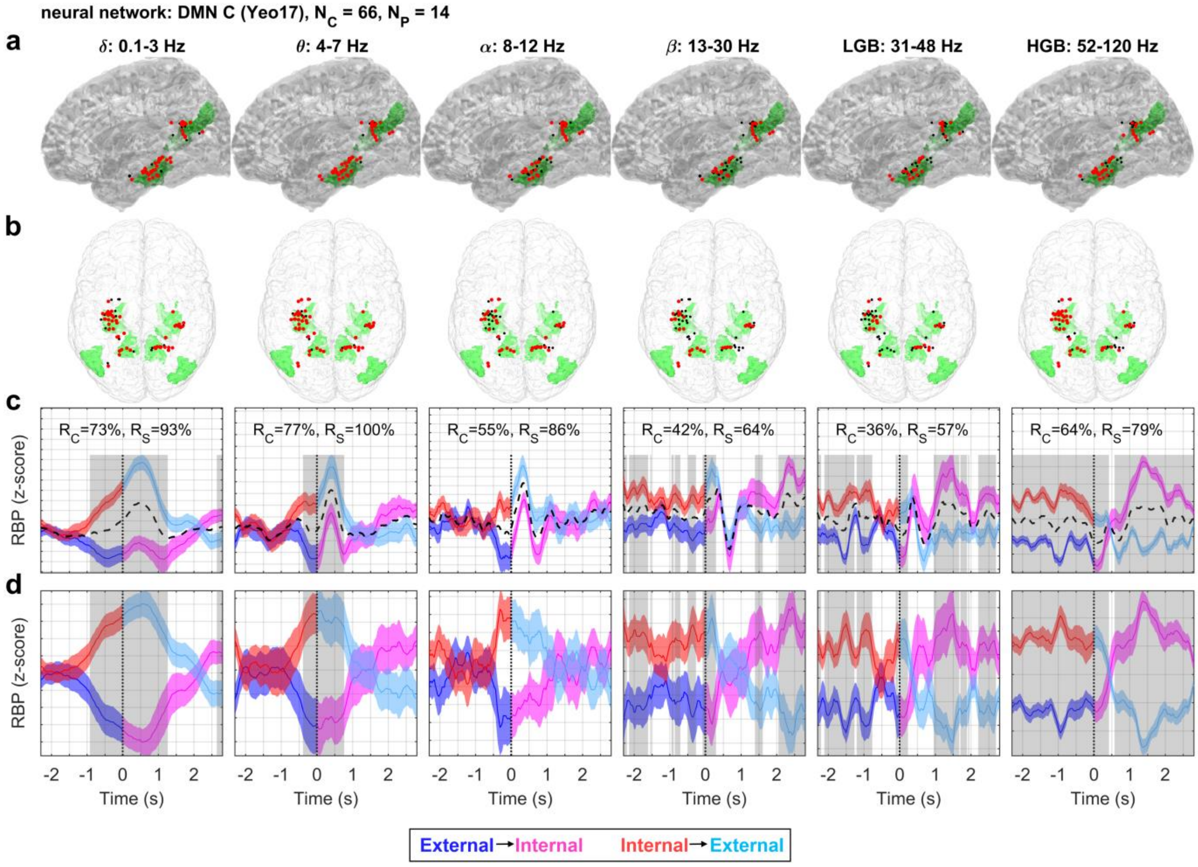

Supplementary Figure S6. The DMN-C during the attention-switching task. Same notations as in Fig. 4. The DMN-C (based on the Yeo-17 brain parcellation, highlighted in green) consisted of 66 channels in 14 subjects. The channels were anatomically distributed mostly in the medial temporal lobe (parahippocampal gyrus, fusiform gyrus, collateral sulcus, and hippocampus) but also in the parietal lobe (angular gyrus and posterior cingulate cortex). The RBP was less smooth, presumably due to the lower number of channels. The activity was consistent with overall DMN behavior only in the HGB. In the LFBs, there was a different activity pattern, most evident in the theta band: a decrease of the LFB power before the presentation of the new stimulus (t = -0.5 s) for the E-task (blue curve) and an increase for the I-task (red curve) shortly before the completion of the self-episodic memory retrieval task. However, these differences were not considered significant on the network level (P > 0.001, FDR corrected). The increase in HGB during the I-task and subsequent switch of the activation during the E-task, suggest the DMN-C activation for the I-task (red and magenta curves) and deactivations/suppressions during the E-task (blue and cyan curves).

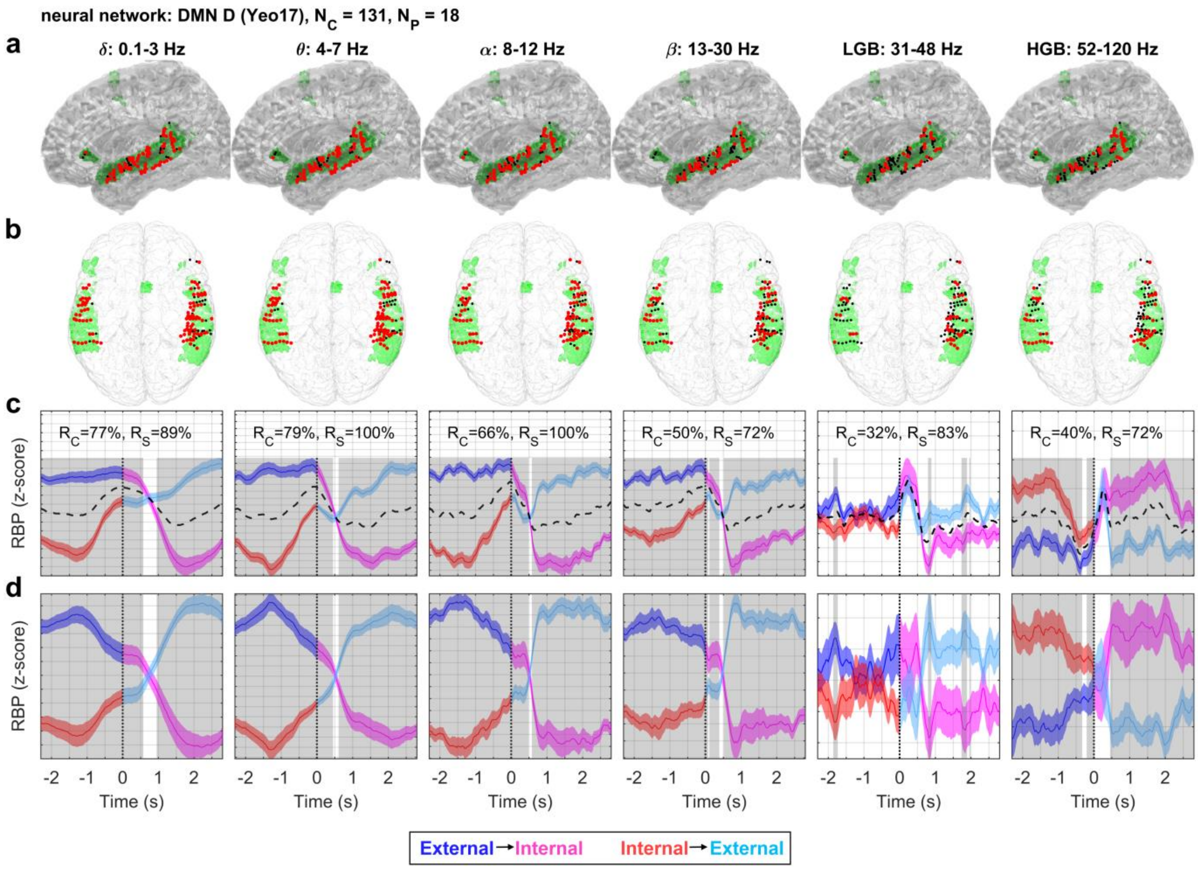

Supplementary Figure S7. The DMN-D during the attention-switching task. Same notations as in Fig. 4. The DMN-D (based on the Yeo-17 brain parcellation, highlighted in green) consisted of 131 channels in 18 subjects. The channels were anatomically distributed dominantly in the temporal lobe (superior and middle temporal gyrus), with a few channels in the frontal lobe (inferior frontal gyrus). Consistent with overall DMN behavior, there was a pronounced difference in the LFBs (< 30 Hz), especially in the alpha band (8–12 Hz) and the opposite activation in the HGB. The relative decrease of the LFB power and increase in the HGB during the I-task and subsequent switch of the activation during the E-task, suggest the DMN-D activation for the I-task (red and magenta curves) and deactivations/suppressions during the E-task (blue and cyan curves).

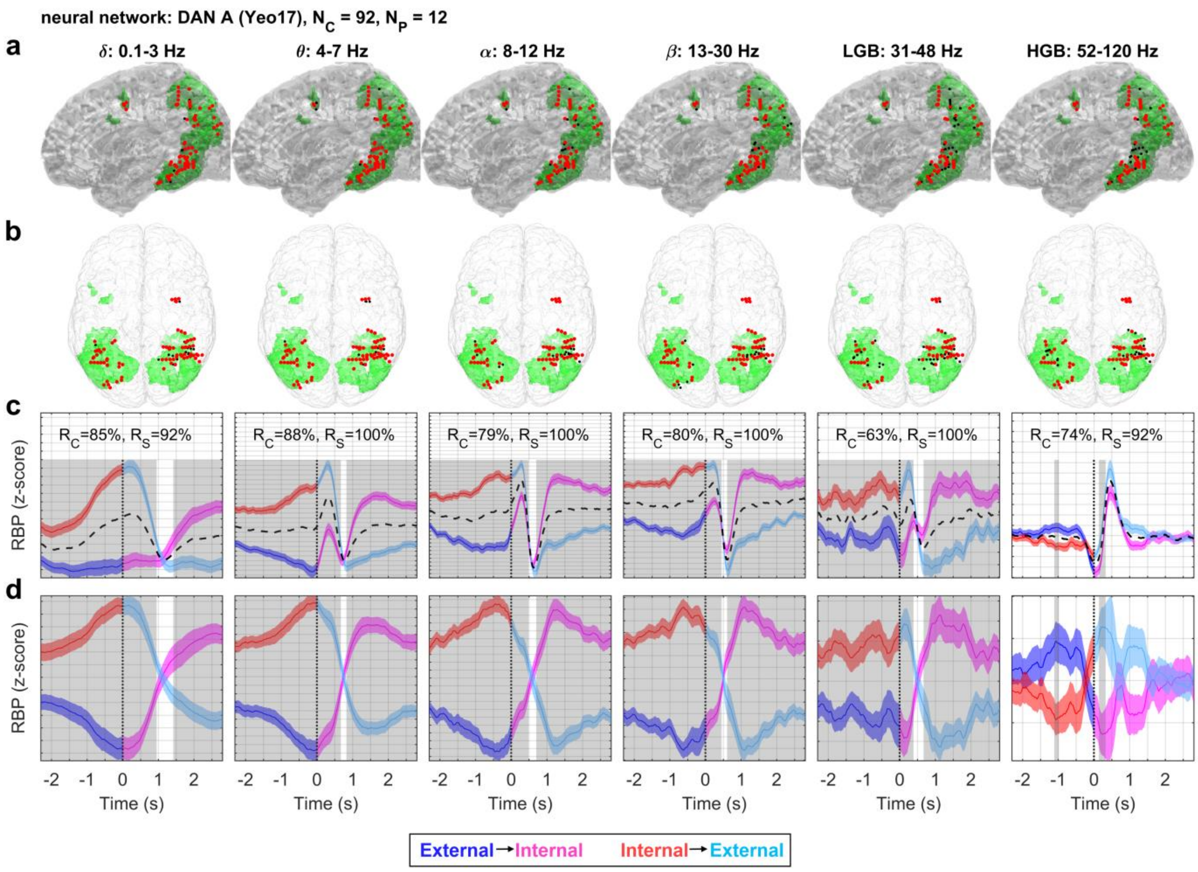

Supplementary Figure S8. The DAN-A during the attention-switching task. Same notations as in Fig. 4. The DAN-A (based on the Yeo-17 brain parcellation, highlighted in green) consisted of 92 channels in 12 subjects. The channels were anatomically distributed dominantly in the temporal lobe (fusiform gyrus, inferior and middle temporal gyrus), parietal lobe (angular gyrus and superior parietal lobule), occipital lobe (middle occipital gyrus) and a few channels in frontal lobe (middle frontal gyrus). The activations in LFB and LGB were consistent with overall DAN behavior. The HGB was dominated by a strong decrease (trough at t = 0 s), likely reflecting DAN-A deactivation during the button press (t = 0 s), followed by a rapid increase (peak at t = 500 ms) of power related to the presentation of the new visual stimulus for both tasks. On the network level, HGB was slightly more activated for the E-task than for the I-task (although the difference was not significant at the significance level P = 0.001, FDR corrected).

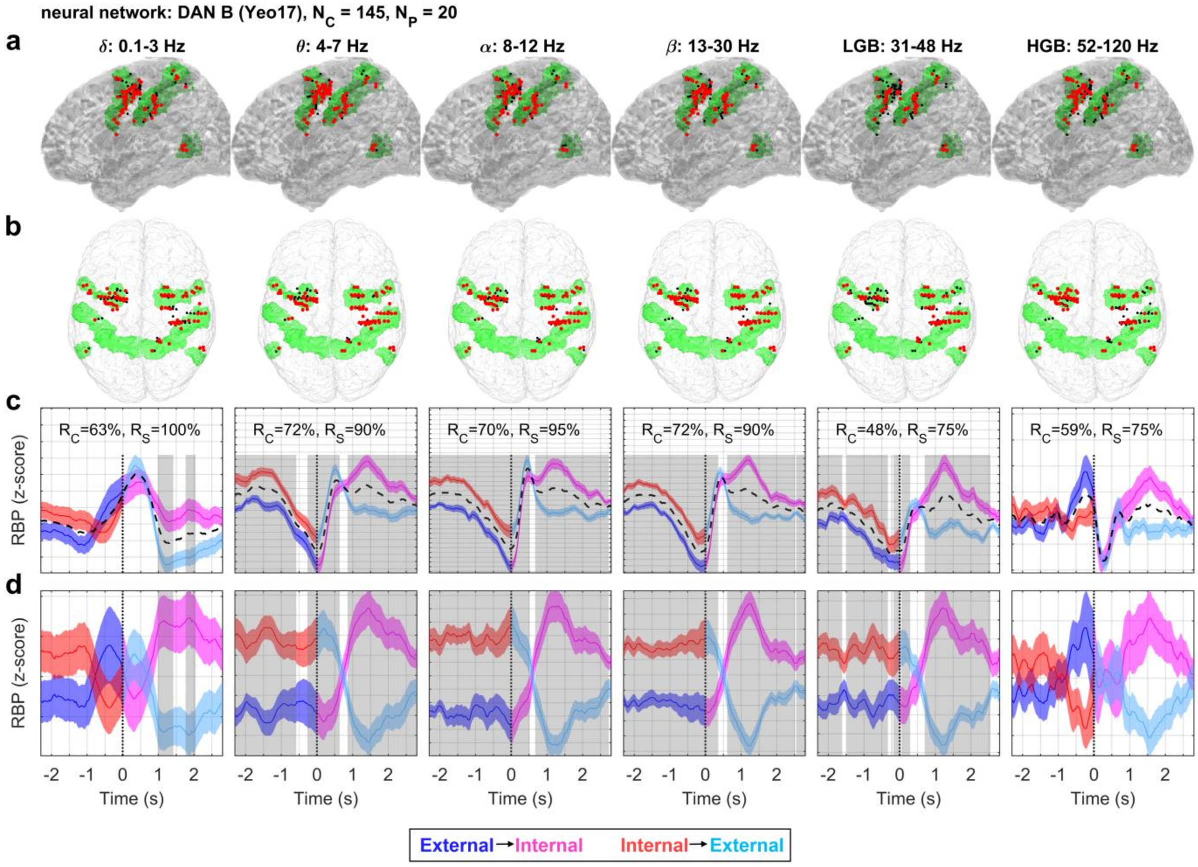

Supplementary Figure S9. The DAN-B during the attention-switching task. Same notations as in Fig. 4. The DAN-B (based on the Yeo-17 brain parcellation, highlighted in green) consisted of 145 channels in 20 subjects. The channels were anatomically distributed dominantly in the frontal lobe (middle and superior frontal gyrus, precentral gyrus), parietal lobe (postcentral gyrus, superior parietal lobule, and supramarginal gyrus) and a few channels in the temporal lobe (middle and inferior temporal gyrus). In the LFBs, there was a strong activity common to both task conditions (black dashed lines), presumably related to the button press and presentation of the new stimulus. After the subtraction of this common activity (bottom row), the polarity of the LFB activations were consistent with the overall behavior of the DAN. The HGB power showed an interesting modulation, with a peak in the E-task shortly before the stimulus presentation (blue curve; t = –250 ms), a rapid decrease (trough at t = 200 ms), followed by a gradual increase, which ceased for the I-task (cyan curve; t = 700 ms), but continued for the E-task (magenta curve' peaking around t = 1.5 s). However, the difference between the task conditions was not significant at the significance level P = 0.001 (FDR corrected).

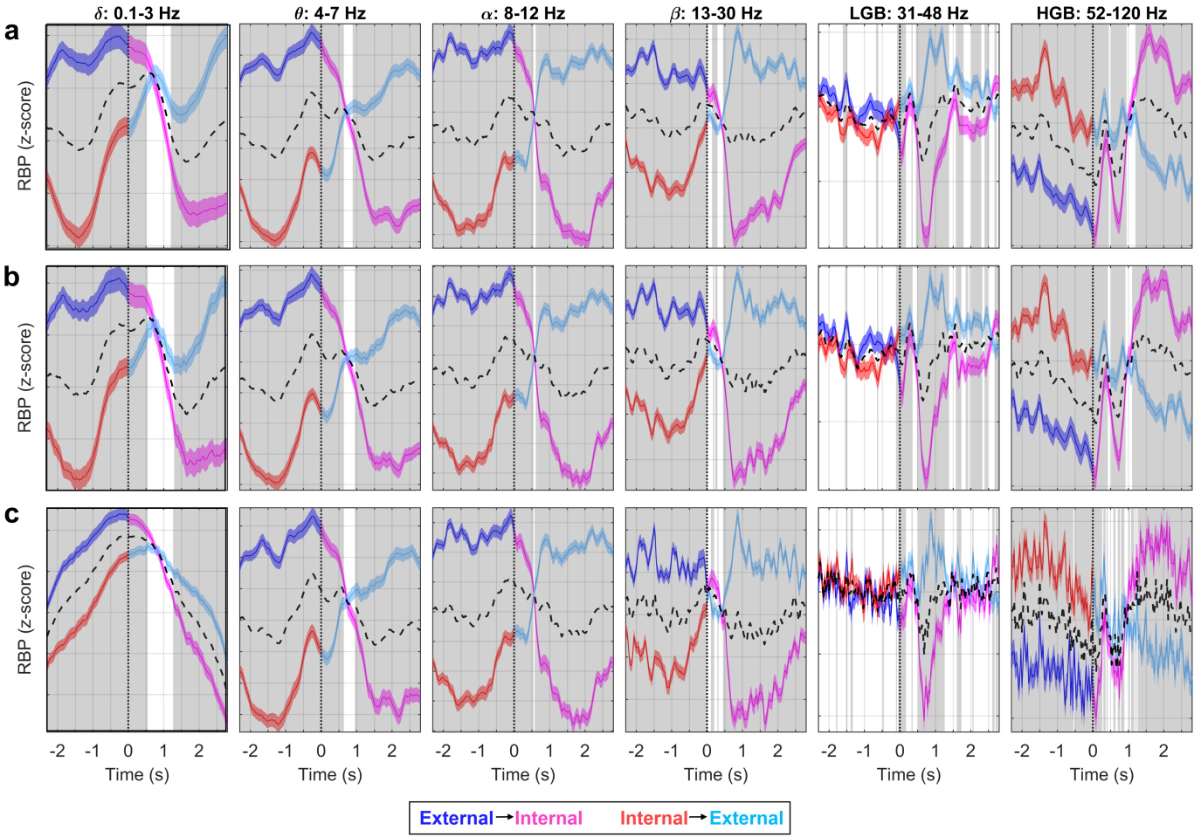

Supplementary Figure S10. Comparisons of RBP estimations for the DMN. Each subplot: Grand average of RBP modulation (y-axis) in time (x-axis) for the external-to-internal (blue–magenta curve) or internal-to-external (red–cyan curve) attention switching conditions computed across all 741 channels from 24 subjects localized to the DMN (plotband: mean ± SEM). The RBP was estimated by **(a)** the STFT without any data rejection, **(b)** the STFT with data rejection (see Methods for more details) and **(c)** the Morlet wavelet transformation. The data rejection had almost no effect on the temporal profiles of the RBP. The Morlet wavelet transform also tightly correlated with temporal profiles of the RBP from STFT in a (correlation coefficient = 0.90 ± 0.05; mean ± SEM across all frequency bands and conditions).

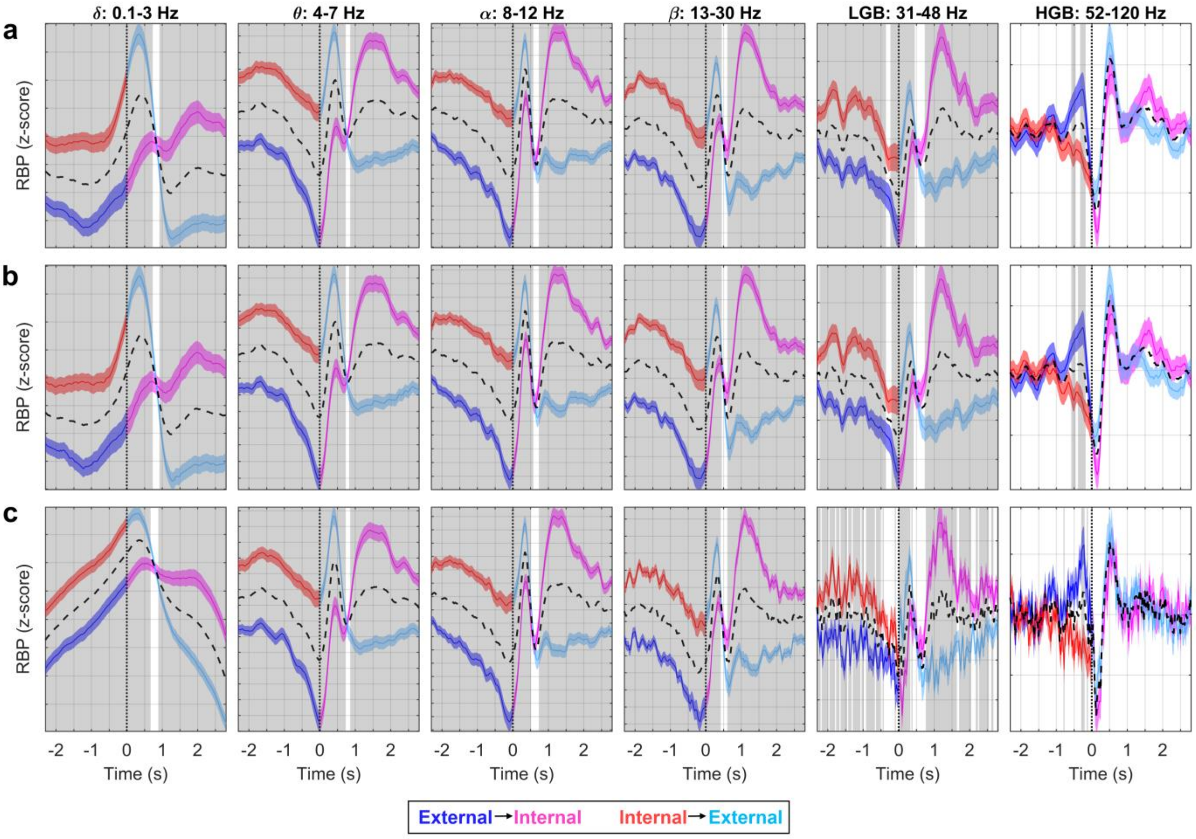

Supplementary Figure S11. Comparisons of RBP estimations for the DAN. All 297 channels from 24 subjects localized to the DAN were used in the computations (same notations as in Suppl. Fig. S4). The RBP was estimated by **(a)** the STFT without any data rejection, **(b)** the STFT with data rejection and **(c)** the Morlet wavelet transformation. The data rejection had almost no effect on the temporal profiles of the RBP. The Morlet wavelet transform also resulted in highly correlated temporal profiles of the STFT-based RBP (correlation coefficient = 0.93 ± 0.03; mean ± SEM across all frequency bands and conditions).

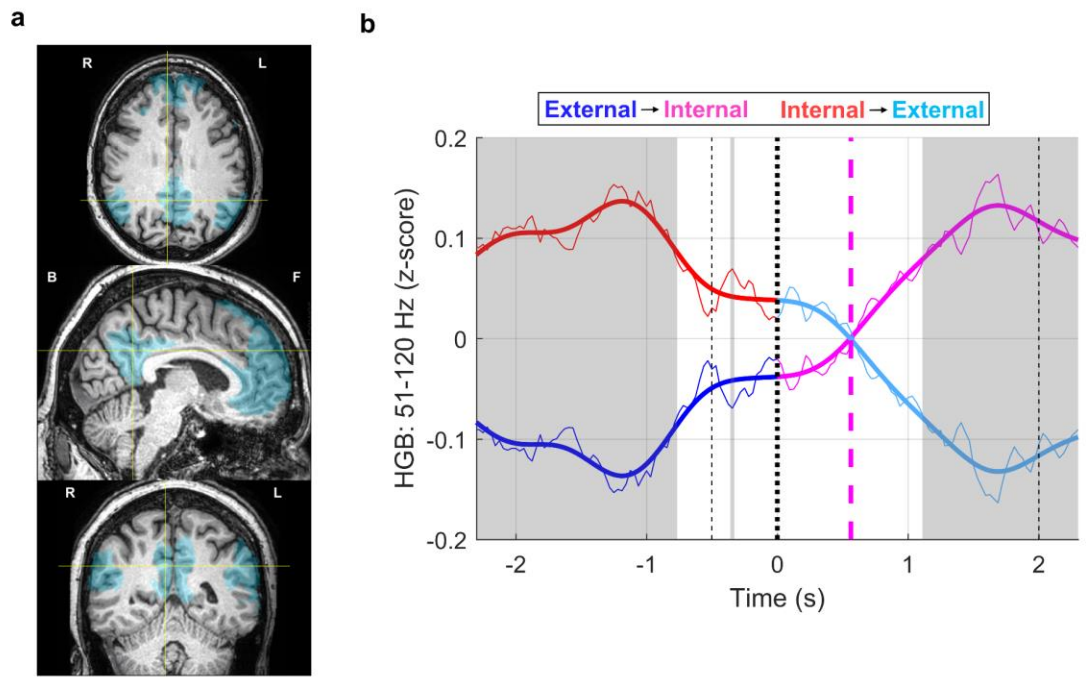

Supplementary Figure S12. Crossover point of HGB activity during the attention-switching task. An exemplary channel (subject P11) located in the posterior cingulate cortex, one of the major nodes of the DMN network, was selected for illustration. **(a)** Pre-implantation MRI (gray-scale) with the DMN highlighted in blue. Yellow crosshairs indicate the position of the selected channel. Top panel: axial view; middle panel: sagittal view; bottom panel: coronal view. R = right, L = left, F = front, B = back. **(b)** Corresponding, trial-averaged HGB power (51–120 Hz) after subtraction of the common (condition non-specific) signal for both task conditions: external–internal attention switching (blue–magenta) and internal–external attention switching (red–cyan). The thick curves are the HGB activity after low-pass (< 1 Hz) filtering, applied to smooth the jittered HGB activity. Gray background indicates significant activity ($P < 0.05$, FDR corrected), where the significance of difference was tested across different trials of the task conditions. Black dotted line indicates the time of the task switch ($t = 0$ s). Magenta dashed line indicates time of the crossover point ($t = 0$ s). We further demanded that the crossover point is located in the time interval $t \in [-0.5, 2]$ s (thin vertical dotted lines).

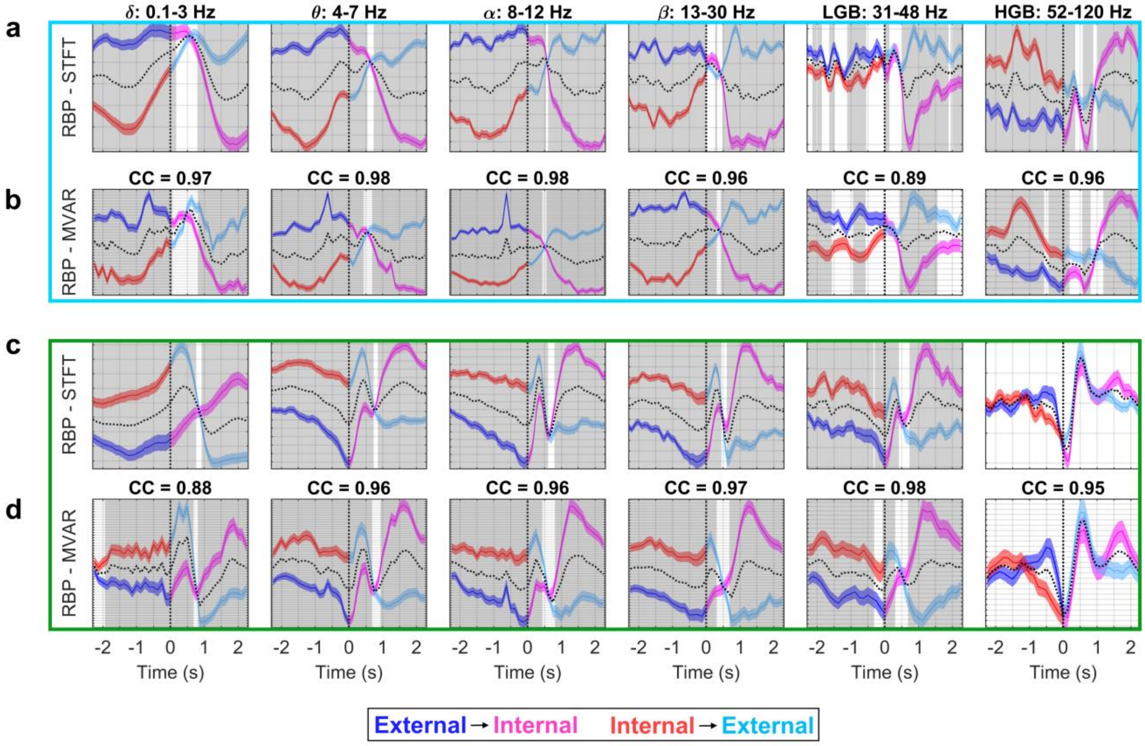

Supplementary Figure S13. Comparison of RBP obtained by two different methods: STFT and an MVAR model. The RBP is shown in the six different frequency bands (columns) indicated by the titles. The RBP was computed as a mean across the iEEG channels of the 17 subjects selected for the functional and effective connectivity presented in Fig. 7. Each subplot: same conventions as in Fig. 4. **(a)** RBP obtained by STFT for the DMN. **(b)** RBP obtained by the MVAR model for the DMN. The title above the second row in each subplot indicates the correlation coefficient (CC) between RBP of the two methods for both task conditions. **(c, d)** Same as in a and b but for the DAN. The overall CC was quite high (0.95 ± 0.01, mean ± SEM, across the six frequency bands and both networks), confirming that the MVAR model was able to capture the major power modulations observed with the more common STFT.

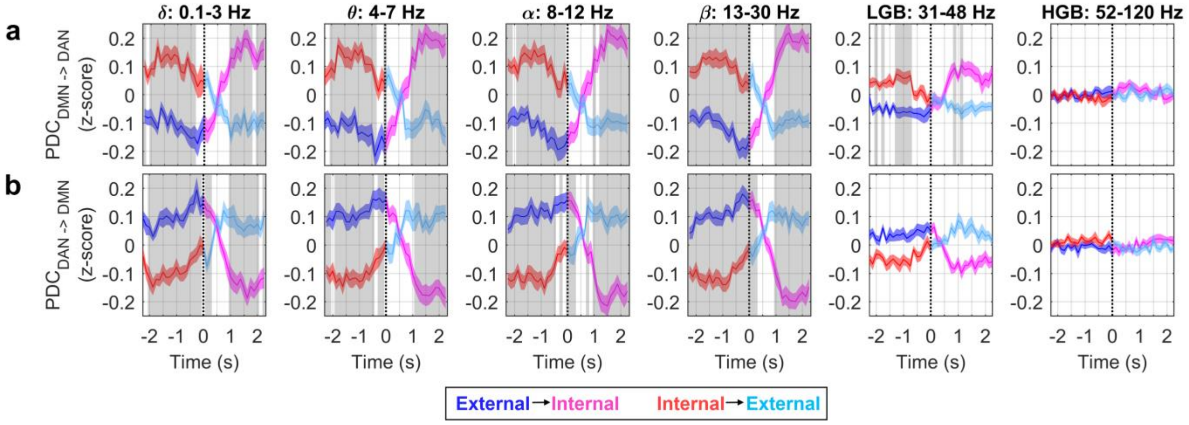

Supplementary Figure S14. Directed functional connectivity estimated by the PDC between the DMN and DAN during attention switching. The PDC (partial directed coherence) based on the MVAR model was computed across 17 subjects. Same notations as in Fig. 7c, d. Each subplot: Grand average of temporally resolved (x-axis) PDC (y-axis) for the external-to-internal (blue–magenta curve) or internal-to-external (red–cyan curve) attention-switching conditions (plotband: mean ± SEM across 17 subjects). Significant differences in distributions between the attention-switching conditions ($P < 0.001$, FDR corrected) marked by gray rectangles. Time t = 0 s (vertical dotted line) represents the time of task switching. The direction of causality is **(a)** from the DMN to DAN and **(b)** from the DAN to DMN. Similar to the DTF (Fig. 7c, d), the PDC showed a clear and significant reversal of the direction of the information flow after the task switch in the low-frequency bands (< 30 Hz), where the influence from the DMN to DAN was higher during the internal attention task and the influence from the DAN to DMN was higher during the external attention task.

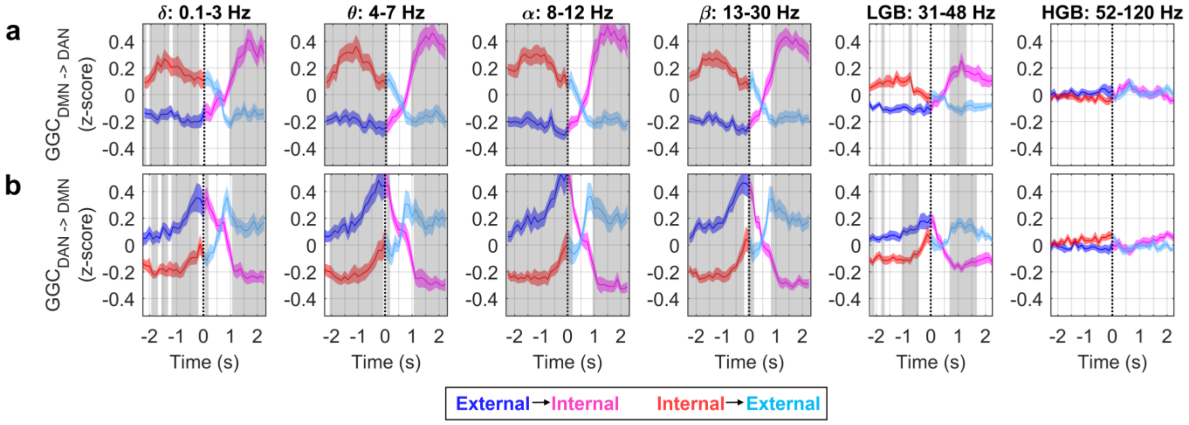

Supplementary Figure S15. Directed functional connectivity estimated by the GGC between the DMN and DAN during attention switching. The GGC (Granger Geweke causality) based on the MVAR model was computed across 17 subjects. Same notations as in Suppl. Fig. 14. The direction of causality is **(a)** from the DMN to DAN and **(b)** from the DAN to DMN. The GGC also showed the reversal of causality after the task switched between the external and internal attention conditions.
