# Supplementary material for: Antagonistic behavior of brain networks mediated by low-frequency oscillations: electrophysiological dynamics during internal–external attention switching
Source: Commun Biol. 2024 Sep 9;7:1105. doi: 10.1038/s42003-024-06732-2 (PMC11385230; doi:10.1038/s42003-024-06732-2)
Supplement: Supplementary file 2 — Reporting summary [file 42003_2024_6732_MOESM2_ESM.pdf]

nature portfolio

Corresponding author(s): Jiri Hammer

Last updated by author(s): Jul 24, 2024

Reporting Summary

Nature Portfolio wishes to improve the reproducibility of the work that we publish. This form provides structure for consistency and transparency in reporting. For further information on Nature Portfolio policies, see our Editorial Policies and the Editorial Policy Checklist.

Statistics

For all statistical analyses, confirm that the following items are present in the figure legend, table legend, main text, or Methods section.

n/a	Confirmed	
☐	☒	The exact sample size (*n*) for each experimental group/condition, given as a discrete number and unit of measurement
☐	☒	A statement on whether measurements were taken from distinct samples or whether the same sample was measured repeatedly
☐	☒	The statistical test(s) used AND whether they are one- or two-sided<br>*Only common tests should be described solely by name; describe more complex techniques in the Methods section.*
☐	☒	A description of all covariates tested
☐	☒	A description of any assumptions or corrections, such as tests of normality and adjustment for multiple comparisons
☐	☒	A full description of the statistical parameters including central tendency (e.g. means) or other basic estimates (e.g. regression coefficient) AND variation (e.g. standard deviation) or associated estimates of uncertainty (e.g. confidence intervals)
☐	☒	For null hypothesis testing, the test statistic (e.g. *F*, *t*, *r*) with confidence intervals, effect sizes, degrees of freedom and *P* value noted<br>*Give P values as exact values whenever suitable.*
☒	☐	For Bayesian analysis, information on the choice of priors and Markov chain Monte Carlo settings
☐	☒	For hierarchical and complex designs, identification of the appropriate level for tests and full reporting of outcomes
☒	☐	Estimates of effect sizes (e.g. Cohen's *d*, Pearson's *r*), indicating how they were calculated

Our web collection on statistics for biologists contains articles on many of the points above.

Software and code

Policy information about availability of computer code

Data collection	A description of data collection (software packages, etc.) is provided in the Methods section (including citations).<br>To wrap up, we used:<br>- PsychToolBox 3 for paradigm implementation and stimuli presentation.<br>- Clinical amplifiers to sample the iEEG data (Natus Quantum).
Data analysis	A description of data analysis toolboxes (software packages, etc.) used in the study is provided in the Methods section (including citations).<br>To wrap up, we used:<br>- Custom scripts for analysis and results visualization publicly available on GitHub (https://github.com/JiriHammer/SEEG_dataAnalysis).<br>- Publicly available brain parcelation from Yeo et al. (2011) available here: https://surfer.nmr.mgh.harvard.edu/fswiki/CorticalParcellation_Yeo2011<br>- TSA package for MVAR models from Schloegl et al. available on GitHub (https://github.com/VisLab/detect/tree/master/TSA).

For manuscripts utilizing custom algorithms or software that are central to the research but not yet described in published literature, software must be made available to editors and reviewers. We strongly encourage code deposition in a community repository (e.g. GitHub). See the Nature Portfolio guidelines for submitting code & software for further information.

Data

Policy information about availability of data

All manuscripts must include a data availability statement. This statement should provide the following information, where applicable:

- Accession codes, unique identifiers, or web links for publicly available datasets
- A description of any restrictions on data availability
- For clinical datasets or third party data, please ensure that the statement adheres to our policy

As the datasets may include sensitive patient-specific information (MRI scans of the head, etc.), we decided not to post them to a publicly open repository. In the manuscript, we inlcuded the following data availability statement:
"The data that support the findings of this study are available from the corresponding author upon reasonable request."

Research involving human participants, their data, or biological material

Policy information about studies with human participants or human data. See also policy information about sex, gender (identity/presentation), and sexual orientation and race, ethnicity and racism.

Reporting on sex and gender	In this study, we did not conduct a sex- or gender-specific analysis. Both male and female patients were treated equally, we made no hypothesis about sex- or gender-specific neural dynamics in the brain here.<br>In the Supplementary table 1, we report on gender (based on self-reporting). Please note, however, that there was no distinction between sex and gender in the patients' cohort (i.e., sex = gender in all 25 cases).
Reporting on race, ethnicity, or other socially relevant groupings	We did not classify people into the different categories (race, ethnicity, social groups, etc). All patients were treated equally.<br>We did not collect this kind of data here.
Population characteristics	The age, duration of the epilepsy of the participants is reported in the Supplementary table 1.
Recruitment	All patients implanted with the intracerebral electrodes were given the opportunity to participate in the study, provided their informed consent and a good health condition and the time of the experiment. We are not aware of any selection bias or other biases that could impact our results.
Ethics oversight	Ethics committee of the Motol University Hospital, Prague, Czech Republic (this information is included in the manuscript Methods section).

Note that full information on the approval of the study protocol must also be provided in the manuscript.

Field-specific reporting

Please select the one below that is the best fit for your research. If you are not sure, read the appropriate sections before making your selection.

☒ Life sciences ☐ Behavioural & social sciences ☐ Ecological, evolutionary & environmental sciences

For a reference copy of the document with all sections, see nature.com/documents/nr-reporting-summary-flat.pdf

Life sciences study design

All studies must disclose on these points even when the disclosure is negative.

Sample size	No sample size calculation was performed. In this study, we report on scarce data of intracranial EEG from epilepsy patients (i.e., using the depth electrodes). Such data are rather rare to acquire (approximately 8 patients per year at our the neurology departments of Motol University Hopsital, Prague, Czech Republic). To provide robust results (i.e., reproducable over several different participants) in a reasonable time determined by the funding projects (typically 3-4 years) , we aimed to include at least 20 different subjects (while it remains a matter of debate if less was also sufficient). In the end, we arrived at 25 subjects. As the reported results were highly reproducible across subjects, we are convinced that the sample size is sufficient.
Data exclusions	Data exclusions (including exclusion criteria) are described in the Methods of the manuscript.<br>To wrap up, we only excluded obviously broken iEEG channels (i.e., data not recording proper intracranial EEG but only/mostly artifacts) and channels in the epilepsy network (due to potentially altered dynamics). The epilepsy network channels were determined by experienced neurologists, blind to the results and analysis of the reported results, solely based on the clinical diagnosis.
Replication	The reported findings were reproduced across 25 subjects (a relatively large cohort given the rarity of intracranial EEG measurements). We tested the significance of difference of the network activity across hundreds of channels (exact numbers detailed in the manuscript). We specifically set the significance threshold very low (P = 0.001) and included false discovery rate correction.
Randomization	Participants were not allocated into different experimental groups. However, the iEEG channels were grouped based on their anatomical localization into the different brain networks (detailed in the manuscript).

Blinding	*Describe whether the investigators were blinded to group allocation during data collection and/or analysis. If blinding was not possible, describe why OR explain why blinding was not relevant to your study.*

Reporting for specific materials, systems and methods

We require information from authors about some types of materials, experimental systems and methods used in many studies. Here, indicate whether each material, system or method listed is relevant to your study. If you are not sure if a list item applies to your research, read the appropriate section before selecting a response.

Materials & experimental systems

n/a	Involved in the study	
☒	☐	Antibodies
☒	☐	Eukaryotic cell lines
☒	☐	Palaeontology and archaeology
☒	☐	Animals and other organisms
☒	☐	Clinical data
☒	☐	Dual use research of concern
☒	☐	Plants

Methods

n/a	Involved in the study	
☒	☐	ChIP-seq
☒	☐	Flow cytometry
☒	☐	MRI-based neuroimaging

Plants

Seed stocks	*Report on the source of all seed stocks or other plant material used. If applicable, state the seed stock centre and catalogue number. If plant specimens were collected from the field, describe the collection location, date and sampling procedures.*
Novel plant genotypes	*Describe the methods by which all novel plant genotypes were produced. This includes those generated by transgenic approaches, gene editing, chemical/radiation-based mutagenesis and hybridization. For transgenic lines, describe the transformation method, the number of independent lines analyzed and the generation upon which experiments were performed. For gene-edited lines, describe the editor used, the endogenous sequence targeted for editing, the targeting guide RNA sequence (if applicable) and how the editor was applied.*
Authentication	*Describe any authentication procedures for each seed stock used or novel genotype generated. Describe any experiments used to assess the effect of a mutation and, where applicable, how potential secondary effects (e.g. second site T-DNA insertions, mosiacism, off-target gene editing) were examined.*
